# Unmasking the Masquerade: A Case Report of Adult-Onset Still's Disease

**DOI:** 10.7759/cureus.83799

**Published:** 2025-05-09

**Authors:** Nicole Haynes, Janina Gregorski

**Affiliations:** 1 Internal Medicine, Walter Reed National Military Medical Center, Bethesda, USA; 2 Emergency Medicine, Walter Reed National Military Medical Center, Bethesda, USA

**Keywords:** adult-onset still’s disease, autoinflammatory disorder, evanescent skin eruption, ferritin, macrophage activation syndrome

## Abstract

Adult-onset Still's disease (AOSD) is a rare, inflammatory condition commonly characterized by polyarthritis, rash, and fever. Its diagnosis is often missed entirely or delayed due to a lack of biomarkers and nonspecific symptoms. We report a case of a 40-year-old female presenting with an unresolving sore throat, small joint arthritis, worsening low-grade fever, and diffuse rash. After an extensive workup requiring multiple hospitalizations, she was ultimately diagnosed with AOSD. Significant symptom improvement was seen following the initiation of canakinumab and a brief course of steroids. Glucocorticoids and disease-modifying antirheumatic drugs are the mainstays of treatment. In those who present with fever of unknown origin and polyarthralgia, once other etiologies have been ruled out, steroids should be administered quickly for symptomatic control while AOSD workup is pursued, to avoid delayed diagnosis. As AOSD is a diagnosis of exclusion, extensive workup is mandatory and requires clinicians to persistently evaluate a plethora of objective data and redirect clinical reasoning to reach the correct diagnosis and avoid complications.

## Introduction

Adult-onset Still’s disease (AOSD) is a systemic inflammatory condition that causes polyarthritis, maculopapular rash, and fever. First described by George Still in 1897 in children, AOSD is diagnosed in those older than 16 years [1]. Its incidence is estimated to be as low as 0.4 cases per 100,000 individuals, with a bimodal peak in the age groups of 15-25 and 36-46 years [1]. The etiology of the disease is poorly understood, but it is suggested to be a reactive syndrome to environmental factors with underlying genetic predispositions [2,3]. Its diagnosis is often delayed or missed altogether due to an overlapping clinical picture with other diseases and its rarity [2]. We present a case report of a female service member undergoing treatment for AOSD.

## Case presentation

**Figure 1:**
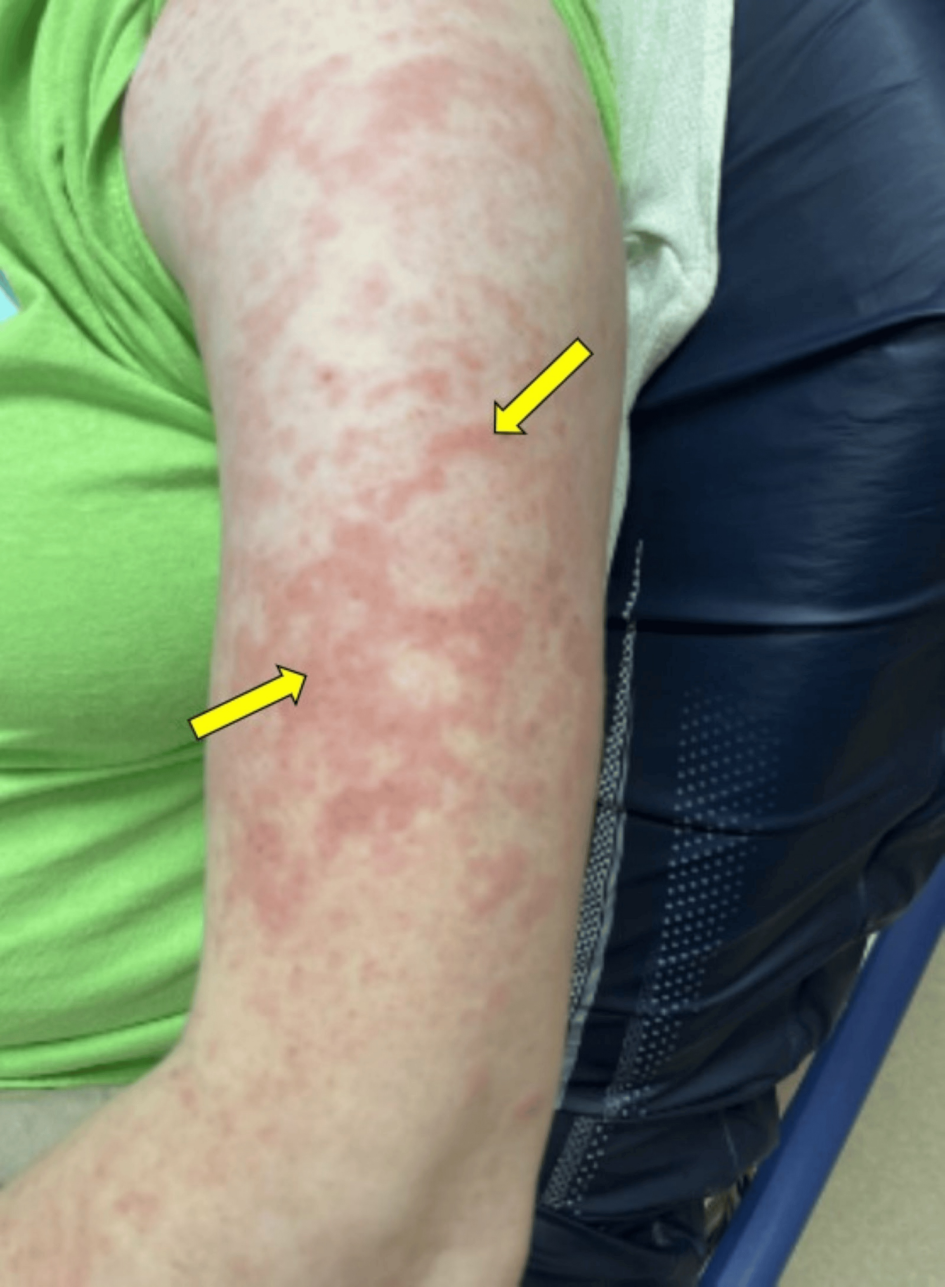
Salmon-colored maculopapular rash seen in AOSD AOSD: adult-onset Still's disease

A 40-year-old female with no significant past medical history presented to the Emergency Department with a day's history of unresolving sore throat, small joint polyarthritis, worsening low-grade fever at home, and diffuse, waxing and waning rash that spared the palms, soles, and face (Figure 1).

She endorsed headache but denied any signs of meningeal irritation. The exam was remarkable for erythematous posterior pharynx without exudate, mild right submental and anterior chain cervical lymphadenopathy, and diffuse rash. The rash appeared urticarial in nature, but the patient had no new exposure to known irritants. There had been recent travel to a southern state earlier in the summer with no reported history of bedbugs, ticks, insect bites, or sick contacts. Further conversation revealed she did have exposure to a domesticated cat and dog. Additional social history revealed a mutually monogamous relationship with the patient’s partner. She was ultimately admitted to internal medicine with elevated CRP levels and recurrent fevers of unknown origin. Workup to this point had been negative for bacteremia, streptococcus PCR, treponemal, urinalysis, respiratory virus, and acute cardiopulmonary findings. Initial medical decision making centered around bacterial versus infectious versus dermatological etiology.

Dermatology was consulted, and they diagnosed viral exanthem, and the patient was scheduled for Infectious Disease follow-up after completing doxycycline 100mg BID for seven days to cover possible tick-borne diseases such as Ehrlichia, Rickettsia, and Arcanobacterium haemolyticum. During this initial hospitalization, the patient remained afebrile with normalizing lab values. On discharge, white blood count and creatinine returned to baseline with mild improvement in her rashes.

Extensive evaluation continued on an outpatient basis, which revealed negative viral sources (HIV, HBSAg, HBcAb, HCV, West Nile virus IgM and IgG, parvovirus PCR, EBV IgM and IgG, CMV IgM and IgG, enterovirus PCR, toxoplasmosis), bacterial (Arcanobacterium haemolyticum culture from throat swab, anti-DNase B, and repeat streptococcus throat culture PCR), other sexually transmitted infections (gonorrhea and chlamydia NAAT of urine, vaginal swab, pharyngeal swab), and tick-borne disease (Rickettsia, Lyme, Ehrlichia, Anaplasma PCR). Although there was some improvement, the patient’s symptoms persisted despite completion of the antibiotic course, with flares of evening fevers accompanied by rash flares. At that time, continued monitoring was planned with repeat labs; however, labs were markedly abnormal: leukocytosis: 13,000/µL with 22% bands, ESR: 50 mm/hr, CRP: 18.2 mg/L, and mild transaminitis (Table 1). The patient was instructed to return to the hospital.

**Table 1:** Laboratory data during workup with peak values listed ALT: alanine aminotransferase; AST: aspartate aminotransferase; CRP: C-reactive protein; ESR: erythrocyte sedimentation rate; WBC: white blood cells

Lab	Value	Reference range (institutional-based)
ESR	54 mm/hr	2.0-37.0 mm/hr
CRP	18.2 mg/L	0.0-0.5 mg/L
AST	1,744 U/L	14.0-50.0 U/L
ALT	1,243 U/L	9.0-52.0 U/L
Ferritin	51,049 ng/mL	13.0-150.0 ng/mL
WBC	13,000/µL	4,800-10,800/µL

**Figure 2:**
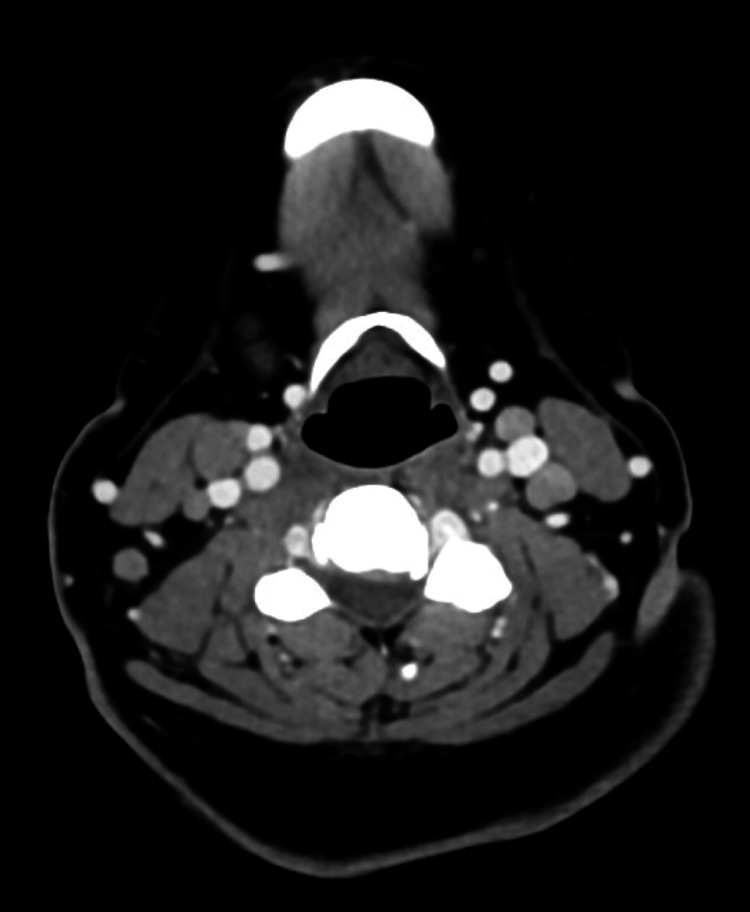
CT neck/soft tissue with contrast showing bilateral anterior and posterior cervical lymphadenopathy CT: computed tomography

**Figure 3:**
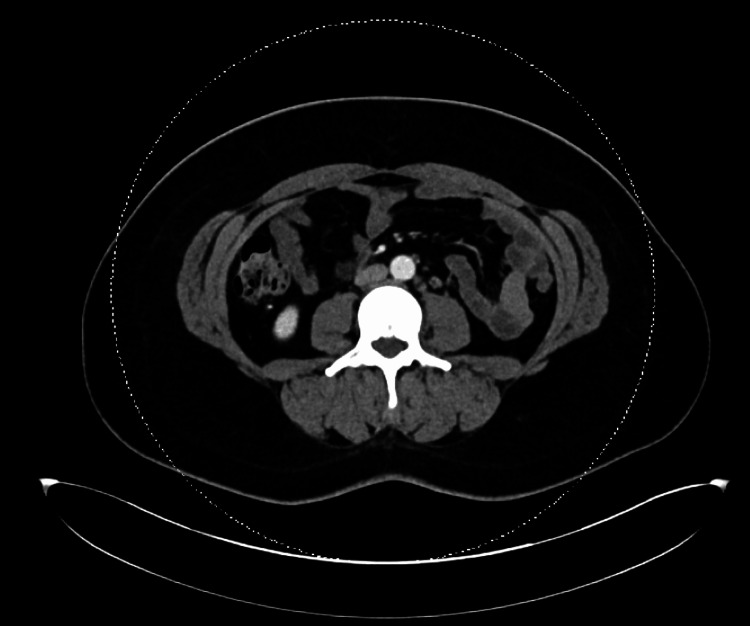
CT CAP with contrast showing scattered retroperitoneal lymphadenopathy CAP: chest, abdomen, and pelvis; CT: computed tomography

**Figure 4:**
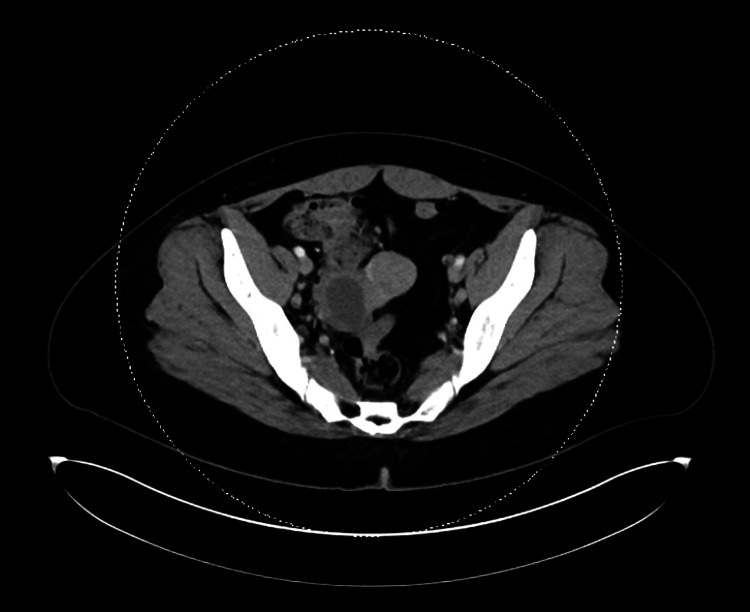
CT CAP with contrast showing right common iliac lymphadenopathy CAP: chest, abdomen, and pelvis; CT: computed tomography

On re-presentation to the Emergency Department, the patient was febrile to 39.4 °C during the evening, which had resolved with otherwise stable vital signs. The exam was notable for right-sided neck fullness, erythematous posterior pharynx, and diffuse maculopapular rash. Presentation was concerning for deep neck space infection versus secondary complication following initial pharyngitis versus continuation of the cause from initial presentation. Workup (Table 1) showed worsening bandemia to 32%, ESR: 54 mm/hr, CRP: 13.6 mg/L, ferritin: 6,185 ng/mL, as well as a negative respiratory panel and chest X-ray. CT neck with contrast showed clustered lateral neck adenopathy but was otherwise without aggressive features. CT of the chest/abdomen/pelvis with contrast was notable for scattered lymphadenopathy involving the mediastinum, bilateral axillary, retroperitoneal, and right common iliac lymph nodes (Figures 2-4).

**Figure 5:**
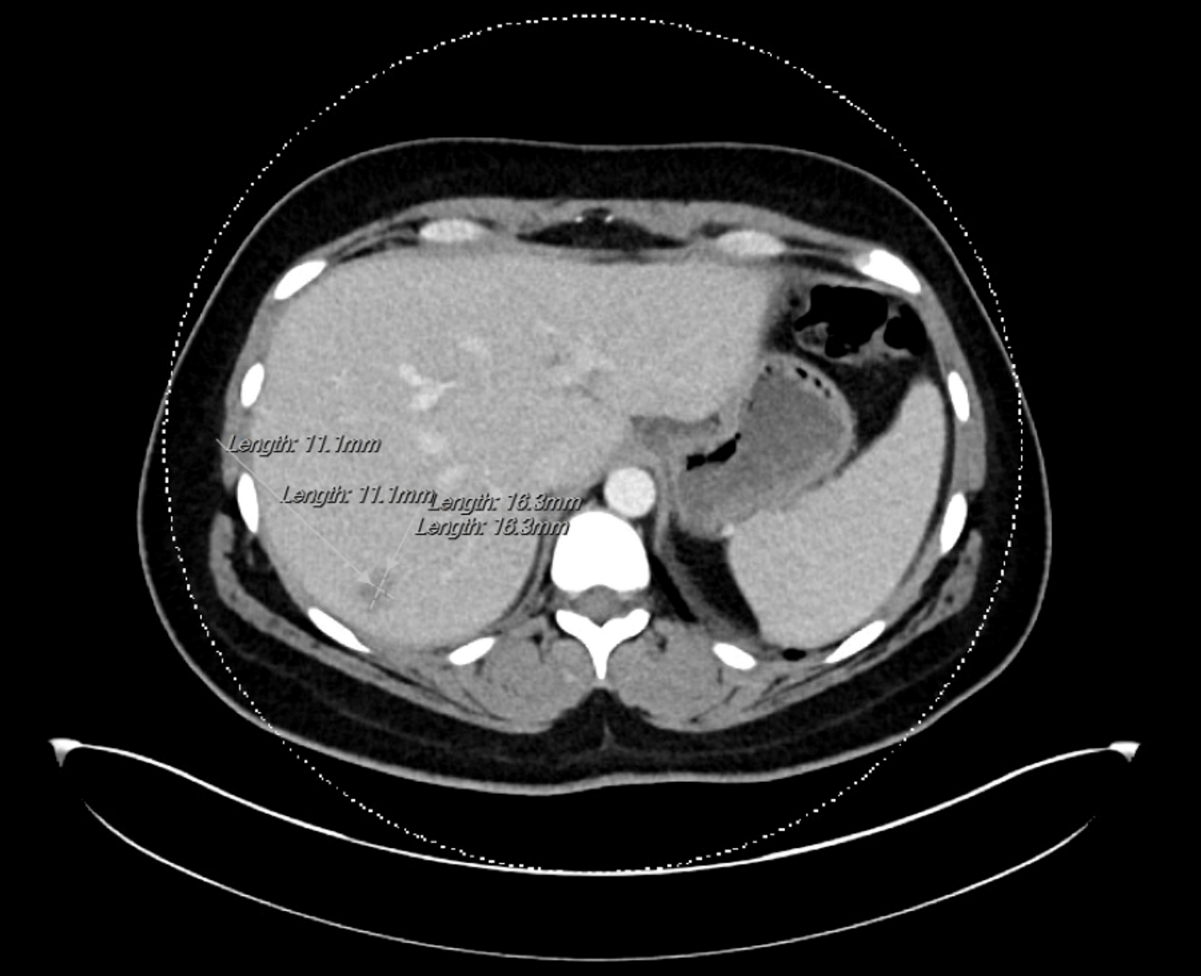
CT CAP with contrast showing a hepatic hypoattenuating lesion measuring 1.3 x 1.6 x 1.1 cm CAP: chest, abdomen, and pelvis; CT: computed tomography

During the second hospital admission, infectious workup continued with negative results for Coxiella, Bartonella, Brucella, Francisella, coccidiomycosis, histoplasma, Blastomyces, cryptococcus, typhi, and blood cultures for 14 days. Dermatology evaluated the patient once more and recommended a rheumatological consult with a plan for an MRI liver. Imaging results showed features suggestive of hemangioma and nonspecific prominent subcentimeter retroperitoneal lymph nodes, but no clear etiology for transaminitis and the overall clinical picture (Figure 5).

Transthoracic echocardiogram was unremarkable with mild mitral regurgitation but otherwise normal anatomy, pressures, and contraction. During this time, her vitals fluctuated with periods of instability that required transfer to the ICU. Concurrently, labs remained significant for rising levels with peak values as follows: AST: 1744 U/L, ALT: 1,243 U/L, and ferritin: 51,049 ng/mL.

The patient's clinical course at this point was highly suggestive of AOSD. She was then started on canakinumab 100 mg IV twice daily and a three-day course of pulse-dosed steroids to treat possible AOSD and hemophagocytic lymphohistiocytosis (HLH), with improvement in symptoms, including resolved fevers and synovitis and downtrending labs. Before discharge, she had an excisional lymph node biopsy and bone marrow biopsy, which ultimately were negative, ruling out lymphoma and leukemia, respectively. A follow-up is planned with Rheumatology and Infectious Disease subspecialties with pending interleukin studies.

## Discussion

AOSD is a rare disease, and there are currently no guidelines regarding the condition in terms of clinical practice. The diagnosis is based on the Yamaguchi criteria [2], which require the presence of several nonspecific features, of which at least two must be major criteria, with a total of five criteria. Major criteria consist of fever greater than or equal to 39 °C that lasts at least one week, arthralgia or arthritis lasting at least two weeks, rash, and/or leukocytosis with at least 80% neutrophils. Minor criteria include sore throat, lymphadenopathy, abnormal liver function tests, and negative antinuclear antibodies.

Given the overlap in clinical signs and symptoms with several diseases, patients with AOSD frequently go unrecognized or misdiagnosed. Setting aside the rarity of the disease, the diagnosis itself is challenging to make as there are no serological markers, genetic histories, or hallmark features unique to the disease. Several biomarkers have been suggested, including ferritin and cytokines, but none have been widely implemented [4]. Our patient was initially misdiagnosed with viral exanthem. Both clinical syndromes are characterized by fever, rash, and arthralgias. Although she had no obvious exposures, it was believed she could have been introduced unknowingly. This line of reasoning is frequently, and rightfully, employed in other cases, but it demonstrates pitfalls in clinicians’ internal diagnostic algorithms. No seromarkers were positive as the workup continued - this was a clue to consider other lesser-known diagnoses early on. Even as she was readmitted, the correct diagnosis was delayed by cervical lymphadenopathy masquerading as a possible deep soft tissue infection.

It should be clearly stated that this disease is not benign. Although it has generally favorable outcomes, if left untreated, it can cause widespread organ damage and, in severe cases, death. Perhaps the most severe complication involves macrophage activation syndrome. The prevalence varies from 10 to 15%, with high mortality rates [1,5].

## Conclusions

Given the lack of specific guidelines to aid direct management, clinicians encountering suspected AOSD find themselves with few evidence-based pathways in the face of an ambiguous clinical syndrome. Treatment is largely empirical, and there is a paucity of options demonstrating robust supporting data. Although research efforts have attempted to find objective markers of the disease, diagnosis requires a compilation of symptoms with extensive workup to exclude mimicking diagnoses. This often creates delays in diagnoses. Providers must be prepared to persistently analyze and redirect clinical reasoning when evaluating patients with fevers of unknown origin. When initial workups remain inconclusive, they must be able to utilize a well-stocked differential toolbox and consider less commonly known diseases like AOSD.
